# Supplementary material for: Identification of photoperiod-induced specific miRNAs in the adrenal glands of Sunite sheep (Ovis aries)
Source: Front Vet Sci. 2022 Jul 22;9:888207. doi: 10.3389/fvets.2022.888207 (PMC9354845; doi:10.3389/fvets.2022.888207)
Supplement: Supplementary file 1 [file Data_Sheet_1.ZIP › Supplementary file/miRDeep2 file.zip!/miRDeep2 file/Novel_Structure/SPLP42A3/Novel_146.pdf]

Provisional ID : NC_019458.2_1338
Score total : 2.3
Score for star read(s) : -1.3
Score for read counts : 0
Score for mfe : 2
Score for randfold : 1.6
Score for cons. seed :
Total read count : 432
Mature read count : 432
Loop read count : 0
Star read count : 0

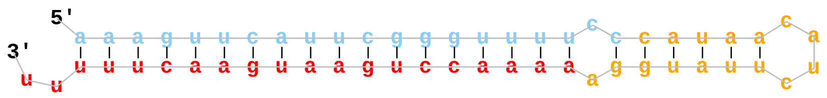

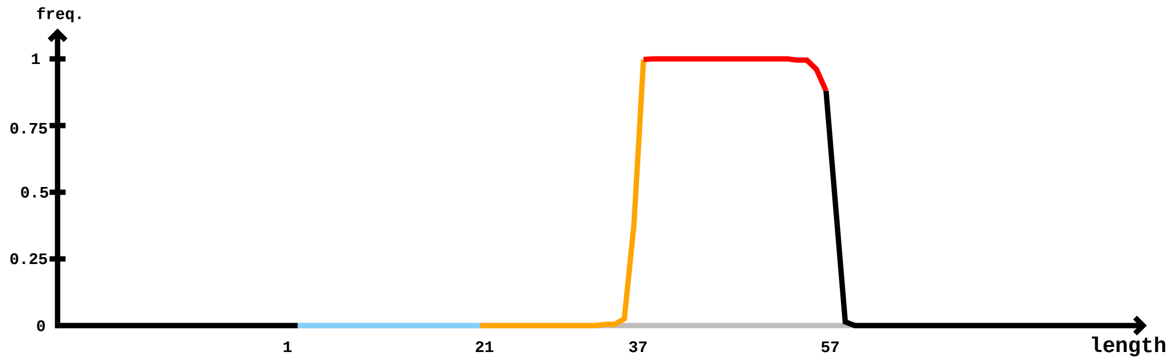

Star Mature

```
5'- gaaagaaguauugcgucggcugaaaaguucauucggguuuucccauaacaucuuauggaaaaaccugaaugaacuuuuuggucaacccaauauaaggaggagacaaagaga   -3'   exp
    .......((((((.((.((((((((((((((((((((((((.((((((....)))))).)))))))))))))))))))))))).)).))))))..................   reads   mm   sample
    .......................................................uggaaaaaccCgaaugaacu....................................   2       1    seq
    .........................................................gaaaaaccCgaaugaacuuu..................................   1       1    seq
    .........................................................gaaaaaccugaaugaacuuuu.................................   4       0    seq
    .........................................................gaaaaaccCgaaugaacuuuu.................................   1       1    seq
    .........................................................gaaaaaccAgaaugaacuuuu.................................   1       1    seq
    .........................................................gaaaaaccugaaCgaacuuuuu................................   1       1    seq
    .........................................................gGaaaaccugaaugaacuuuuu................................   1       1    seq
    ..........................................................aaaaaccCgaaugaacuuu..................................   1       1    seq
    ..........................................................aaaaaccugaaugaacCuu..................................   1       1    seq
    ..........................................................aaaaaUcugaaugaacuuu..................................   12      1    seq
    ..........................................................aaaaaccugaaGgaacuuuu.................................   1       1    seq
    ..........................................................aaaaaUcugaaugaacuuuu.................................   26      1    seq
    ..........................................................aaaaaccugaaugaacCuuuu................................   1       1    seq
    ..........................................................aaaaaUcugaaugaacuuuuu................................   78      1    seq
    ..........................................................Gaaaaccugaaugaacuuuuu................................   1       1    seq
    ..........................................................aaaaaccugaaCgaacuuuuug...............................   3       1    seq
    ..........................................................aaaaaUcugaaugaacuuuuug...............................   24      1    seq
    ..........................................................aaaaaccCgaaugaacuuuuug...............................   2       1    seq
    ..........................................................aaaaaccugaaugaacCuuuug...............................   1       1    seq
    ..........................................................aaaaaccugaaugaacuuuuugg..............................   2       0    seq
    ...........................................................aaaaccugaaugGacuuuu.................................   1       1    seq
    ...........................................................aaaaccAgaaugaacuuuu.................................   1       1    seq
    ...........................................................aaaCccugaaugaacuuuuu................................   1       1    seq
    ...........................................................aaaaUcugaaugaacuuuuu................................   247     1    seq
    ...........................................................aaaaccAgaaugaacuuuuu................................   2       1    seq
    ...........................................................aaaaccugaaugaacuuuuu................................   8       0    seq
    ...........................................................aaaCccugaaugaacuuuuug...............................   2       1    seq
    ...........................................................Gaaaccugaaugaacuuuuug...............................   1       1    seq
    ...........................................................aaaaccugaaugaacuuuuuU...............................   2       1    seq
    ...........................................................aaaaccugGaugaacuuuuugg..............................   1       1    seq
    ...........................................................Gaaaccugaaugaacuuuuugg..............................   2       1    seq
    ...........................................................aaaaccGgaaugaacuuuuugg..............................   1       1    seq
    ............................................................aaaccAgaaugaacuuuuug...............................   1       1    seq
```
